# *Mycobacterium lentiflavum* Infection in Immunocompetent Patient

**DOI:** 10.3201/eid1101.040523

**Published:** 2005-01

**Authors:** Chiara Molteni, Lidia Gazzola, Miriam Cesari, Alessandra Lombardi, Franco Salerno, Enrico Tortoli, Luigi Codecasa, Valeria Penati, Fabio Franzetti, Andrea Gori

**Affiliations:** * University of Milan, Milan, Italy; † Careggi Hospital, Florence, Italy; ‡ Villa Marelli Institute, Milan, Italy

**Keywords:** Mycobacterium lentiflavum, nontuberculous mycobacteria, tuberculosis, dispatch

## Abstract

*Mycobacterium lentiflavum* is a recently described nontuberculous mycobacterium that has mainly clinical importance in young children with cervical lymphadenitis and in immunocompromised patients. We describe a case of chronic pulmonary infection in an immunocompetent patient. Our observation confirms clinical, diagnostic, and treatment difficulties in the management of *M. lentiflavum* infection.

*Mycobacterium lentiflavum* was described as a nontuberculous mycobacterium in 1966 (*1*,*2*). Most of the isolates represented fortuitous isolations, although recently its identification has posed concerns about its possible clinical importance. *M. lentiflavum* was mainly isolated from lymph nodes of young children, while isolations from other sites (lung specimens included) were described only in immunocompromised patients (*3*–*9*).

We describe, for the first time, a chronic pulmonary *M. lentiflavum* infection in an elderly immunocompetent woman. Our report confirms the emergence of this nontuberculous mycobacteria infection in immunocompetent patients and underlines the clinical, diagnostic, and therapeutic difficulties in its management.

## The Study

In February 2000, dyspnea, productive cough, hemoptysis, weight loss, weakness, and slight fever developed in a 67-year-old woman with a previous diagnosis of pulmonary tuberculosis (47 years earlier). A chest radiograph showed post-tuberculous fibrodystrophy of the right upper lobe. Ziehl-Neelsen smear gave positive results, and a nontuberculous mycobacterium was isolated. Drug-susceptibility tests, performed on agar medium by proportion method, showed sensitivity to rifampin, ethambutol, and pyrazinamide and resistance to streptomycin and isoniazid. Treatment with isoniazid, pyrazinamide, ethambutol, and rifampin was begun for 3 months without any microbiologic changes. The persistence of acid-fast bacilli and nontuberculous *Mycobacterium*–positive cultures in the sputum were interpreted as a chronic nontuberculous mycobacterial colonization not associated with true pathogenic damage, and no further treatment was undertaken.

In March 2002, the patient was admitted to our hospital because of productive cough, weakness, dyspnea, hemoptysis, fever, and weight loss. Radiograph and computed tomography scan showed worsening chest abnormalities, with the appearance of a widespread reticulonodular alteration and an opacity in the left middle lobe (Figure A).

**Figure:**
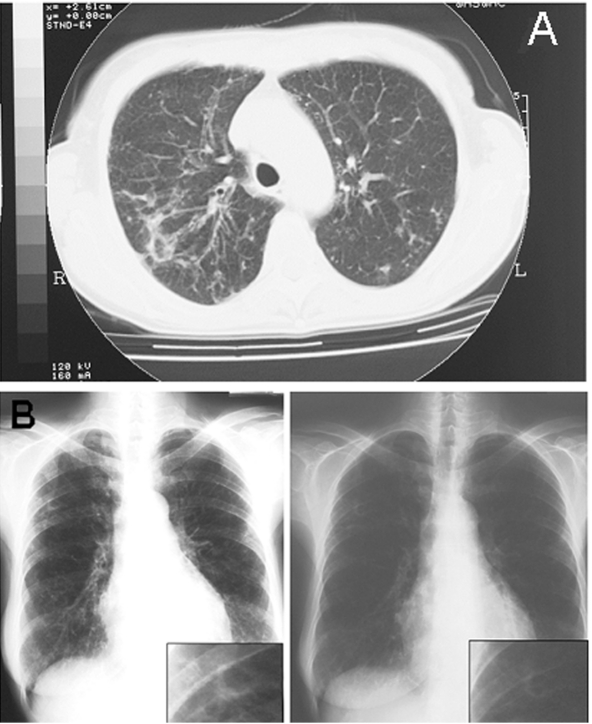
A) Pulmonary computed tomographic scan representation of *Mycobacterium lentiflavum* lesions. Radiologic image shows the appearance of a widespread reticulonodular alteration and an opacity in the left middle lobe. B) Chest radiograph evolution after 3 months of treatment shows a sustained improvement of the radiologic alterations to the left pulmonary middle lobe.

The sputum smear was still positive for acid-fast bacilli (polymerase chain reaction [PCR] specific for *M. tuberculosis* and *M. avium* DNA was negative), and routine cultures for mycobacteria yielded unidentified scotochromogenic mycobacterium. HIV test was performed to investigate a possible cause of immune impairment, but it was negative. Lymphocyte subsets by flow cytometry were studied and showed normal values. In addition, a killing test to evaluate macrophage activity was performed, and a diagnosis of chronic granulomatosis disease was excluded. Concomitantly, cultures from sputum and from broncholavage were performed for either standard bacteria or fungi (including *Pneumocystis carinii*). These cultures did not identify other pathogens. Serologic tests to detect *Chlamydia pneumoniae*, *Mycoplasma pneumoniae*, and *Legionella* species (including urinary antigens) were performed and produced negative results, thus confirming the pathogenic role of the *Mycobacterium lentiflavum*.

Susceptibility tests, using proportion methods, showed sensitivity to clarithromycin, ethambutol, isoniazid, streptomycin, rifabutin, cycloserine, and terizidon and resistance to rifampin, amikacin, kanamycin, pyrazinamide, and ofloxacin. According to the clinical history, microbiologic results, and susceptibility pattern (clarithromycin MIC 2 μg/mL), treatment with clarithromycin was initiated. The patient was released after 10 days with no fever; a slight, yet progressive, improvement of radiologic features; and a substantial recovery of the clinical conditions.

All conventional identification procedures, including cultural, biochemical, and enzymatic tests, failed to properly identify the species. Moreover, many questions remained unresolved, and further clarification was needed about the origin and effect of treatment on the clinical response. Because of the need to reach a definitive diagnosis, we sent the unidentified *Mycobacterium* culture to one of the Italian reference laboratories for mycobacteria.

Two months after hospitalization, the diagnosis of *M. lentiflavum* was obtained by analyzing cell wall mycolic acids by using high-performance liquid chromatographic test and by nucleic acid sequence analysis of PCR-amplified 16S ribosomal RNA gene fragments. After 3 months of clarithromycin treatment, the patient completely recovered, and chest radiograph showed sustained improvement (Figure B). Mycobacterial investigations, which had produced negative sputum samples after 1 month of treatment, once again gave positive results. After a new evaluation of drug susceptibility that showed no change in drug-resistance pattern, a further treatment with clarithromycin, ethambutol, rifabutin, and ciprofloxacin was undertaken, but it was prematurely ended because of poor patient compliance.

At present, after a 3-year follow up, the patient complains of intermittent hemoptysis, weakness, and dyspnea. Radiographic examinations still show the known widespread reticulonodular alterations, and sputum cultures are persistently positive for acid-fast bacilli.

## Conclusions

*M. lentiflavum* is a recently described nontuberculous mycobacterium (*1*,*2*). Most isolates have represented fortuitous isolations that required critical evaluation about their clinical importance. Indeed, as summarized in the Table, *M. lentiflavum* identification has been shown to cause disease in only few cases. All of these cases were described in Europe. Most reports describe isolates from cervical lymphadenitis of very young children (*3*–*9*); other anatomic sites are less frequently implicated (*1*,*7*,*8*). The few *M. lentiflavum* pulmonary cases were described in immunocompromised patients only (*5*,*8*,*9*).

**Table:** Summary of clinical features for 14 patients with *Mycobacterium lentiflavum* infection*

Patient no. (ref. no.)	Age	Sex	Concomitant disease	Intercurrent treatment	Side of infection	Susceptibility test	Antimycobacterial therapy	Clinical outcome
1 (*3*)	19 mo	M	No	No	Cervical lymph node	No	Surgical excision	Recovery (resolved)
2 (*4*)	42 mo	M	No	No	Cervical lymph node	No	Surgical excision	Recovery
3 (*4*)	33 mo	M	No	No	Cervical lymph node	No	Surgical excision	Recovery
4 (*2*)	6 y	F	No	No	Cervical lymph node	ND	Rif, clm/3 wk surgical excision	Recovery
5 (*2*)	4 y	F	ND	ND	Cervical lymph node	ND	Inh, rif/† surgical excision	Recovery
6 (*2*)	4 y	M	ND	ND	Cervical lymph node	ND	surgical excision	Recovery
7 (*6*)	3 y	M	No	No	Cervical lymph node	ND	Clm, eth/6mo	Persistent suppuration
8 (*I7*)	52 y	F	Antisynthetase syndrome	Corticosteroid	Synovial fluid of wrist	inh R, rif R, str R, eth R, pza R, cys S	inh, rif, eth, pza/† fus, levo, clm/1wk	Exitus
9 (*8*)	49 y	M	HIV infection	HAART	Blood, lung	clm S, rib S	clm, rib, eth/4mo	Recovery
10 (*1*)	85 y	F	Diabetes mellitus	ND	Thoracic vertebrae	No	inh, rif, pza/3mo Inh, rif/6mo	Improvement
11 (*2*)	58 y	M	Rheumatoid arthritis	Corticosteroid	Lung	ND	inh, rib, eth, pza/4mo	No improvement
12 (*2*)	61 y	F	COPD, ovarian carcinoma	Reiterated chemotherapy	Lung	ND	rif, inh, pza/† rib, eth, clm, cip/†	No improvement (unchanged)
13 (*2*)	45 y	M	HIV infection, NHL	HAART	Hepatic nodular lesion	ND	rib, clm, eth, cip/2 mo Rib, clm/4mo	Recovery
14 (Molteni)	70 y	F	COPD, lung fibrodystrophy	No	Lung	inh R, str R, rif R, amik R, km R, pza R, oflox R, clm S, eth S, cys S, ter S, rib S	cip, inh/1mo inh, pza, eth, rif/3mo clm/3mo clm, eth, rib, cip/2wk	No improvement

*M, male; F, female; ND, not done; COPD, chronic obstructive pulmonary disease; HAART, higly active antiretroviral therapy; amik, amikacin; clm, clarithromycin; cys, cycloserine; eth, ethambutol, fus, fusidic acid; inh, isoniazid; km, kanamycin; levo, levofloxacin; oflox, ofloxacin, pza, pyrazinamide; rib, rifabutin; rif, rifampin, str, streptomycin; ter, terizidon. †Treatment duration not determined.

We describe, for the first time, a chronic pulmonary infection due to *M. lentiflavum* in an immunocompetent patient. Our observation provides further evidence that this species should be added to the growing list of nontuberculous mycobacteria, which can cause pulmonary disease in both immunocompromised and immunocompetent patients.

Traditional identification techniques are widely insufficient in providing a correct diagnosis, and more sophisticated diagnostic methods need to be improved. Susceptibility tests have reliability problems.

When the difficulties in reaching a diagnosis are considered, the identification standard techniques, based on the rate of growth, pigmentation, and biochemical tests, even if well established and relatively inexpensive, are unable to identify most nontuberculous mycobacteria. Laboratory methods that perform better, such as high-performance liquid chromatography and genetic investigations of the 16S rRNA gene fragments, through the use of nucleic acid probes, sequencing, and amplification, have to be added to diagnostic protocols for nontuberculous mycobacteria diagnosis. Moreover, new reference centers should be created and organized. Cooperation programs among research laboratories, which have provided a structured experience with complex methods, have to be implemented, and an effective collaboration should be created.

In addition to the diagnostic uncertainty, interpreting the sensitivity tests is also of concern. Information provided by tests performed on solid medium was discordant and created serious misunderstanding in the therapeutic choices. Thus, in the presence of nontuberculous mycobacteria, the susceptibility profile must be performed on liquid media, and tests on agar by proportion method should be avoided because of the risk of obtaining false-resistant results. Because of the difficulties in executing these methods and interpreting their results, we recommend that the tests be performed by experienced laboratories that can maintain feedback for both peripheral laboratories and clinicians.

In 1998, the Italian National Institute of Health launched, in accordance with the World Health Organization and International Union Against Tuberculosis and Lung Disease, a project to implement proficiency testing; results from the first (1998–1999) and second surveys (2000) showed substantial improvement in the accuracy of drug-susceptibility testing in the network. These data demonstrate that establishing and coordinating a focused monitoring program were effective in improving the quality of drug susceptibility results (*10*).

By describing a recurrent chronic pulmonary infection, our report provides further evidence of the possible role of *M. lentiflavum* as an emerging human pathogen. Our data also stress the diagnostic and therapeutic difficulties in the management of this nontuberculous *Mycobacterium* infection.
